# Dynamics of the surgical microbiota along the cardiothoracic surgery pathway

**DOI:** 10.3389/fmicb.2014.00787

**Published:** 2015-01-13

**Authors:** Sara Romano-Bertrand, Jean-Marc Frapier, Brigitte Calvet, Pascal Colson, Bernard Albat, Sylvie Parer, Estelle Jumas-Bilak

**Affiliations:** ^1^Equipe Pathogènes et Environnements, UMR 5119 ECOSYM, Université Montpellier 1Montpellier, France; ^2^Département d'Hygiène Hospitalière, Centre Hospitalier Régional Universitaire de MontpellierMontpellier, France; ^3^Service de Chirurgie Thoracique et Cardiovasculaire, Centre Hospitalier Régional Universitaire de MontpellierMontpellier, France; ^4^Département de Réanimation de Chirurgie Cardiothoracique, Centre Hospitalier Régional Universitaire de MontpellierMontpellier, France

**Keywords:** microbial diversity, temporal temperature gel electrophoresis, surgical wound, dysbiosis, pathobiome, *Propionibacterium*, *Staphylococcus*, *Proteobacteria*

## Abstract

Human skin associated microbiota are increasingly described by culture-independent methods that showed an unexpected diversity with variation correlated with several pathologies. A role of microbiota disequilibrium in infection occurrence is hypothesized, particularly in surgical site infections. We study the diversities of operative site microbiota and its dynamics during surgical pathway of patients undergoing coronary-artery by-pass graft (CABG). Pre-, per-, and post-operative samples were collected from 25 patients: skin before the surgery, superficially and deeply during the intervention, and healing tissues. Bacterial diversity was assessed by DNA fingerprint using 16S rRNA gene PCR and Temporal Temperature Gel Electrophoresis (TTGE). The diversity of Operational Taxonomic Units (OTUs) at the surgical site was analyzed according to the stage of surgery. From all patients and samples, we identified 147 different OTUs belonging to the 6 phyla *Firmicutes*, *Actinobacteria*, *Proteobacteria*, *Bacteroidetes*, *Cyanobacteria*, and *Fusobacteria*. High variations were observed among patients but common themes can be observed. The *Firmicutes* dominated quantitatively but were largely encompassed by the *Proteobacteria* regarding the OTUs diversity. The genera *Propionibacterium* and *Staphylococcus* predominated on the preoperative skin, whereas very diverse *Proteobacteria* appeared selected in peri-operative samples. The resilience in scar skin was partial with depletion in *Actinobacteria* and *Firmicutes* and increase of Gram-negative bacteria. Finally, the thoracic operative site presents an unexpected bacterial diversity, which is partially common to skin microbiota but presents particular dynamics. We described a complex bacterial community that gathers pathobionts and bacteria deemed to be environmental, opportunistic pathogens and non-pathogenic bacteria. These data stress to consider surgical microbiota as a “pathobiome” rather than a reservoir of individual potential pathogens.

## Introduction

In the last decade, studies of human associated microbiota have increased our knowledge about host-bacteria interactions (Pflughoeft and Versalovic, 2012) and their consequences in health and diseases (Kuczynski et al., 2012). Colonizing all cutaneous and mucosal body sites and interacting with our immune system (Morgan and Huttenhower, 2012), microbiota are an important part of our organism considered as our second genome (Grice and Segre, 2011) encoding for a supplementary organ (Lederberg and McCray, 2001; Human Microbiome Project Consortium, 2012). The links between microbiota disequilibrium, named dysbiosis, and diseases (Turnbaugh et al., 2009; Zeeuwen et al., 2012) are increasingly described and enrich the concept of supplementary organ.

Health-care associated infections (HAI) are caused by opportunistic bacteria that often belong to the endogenous microbiota of patients and benefit from the weakness of their host to provoke infectious diseases. HAI occurred after medical cares, which drive opportunistic pathogen's emergence and infection. It is probable that bacterial adaptation and selection linked to microbiota disequilibrium are involved in HAI rather than true bacterial virulence.

Particularly, surgical procedures deal directly with the microbiota of the target organ and of the skin at the surgical site, inducing local and systemic inflammation associated with antiseptic and antibiotic pressures (Jakob and Stanga, 2010). The balance of cutaneous microbiota is particularly sensitive to environmental variations (Grice and Segre, 2011) and one can hypothesize that surgical procedures may upset the ecological balance at the surgical site.

Surgical site infections (SSIs) are part of the more common health-care associated infections (HAIs) after urinary tract and pulmonary infections, and among the major concerns of surgeons (Rosenthal et al., 2013). In cardiac surgery, severe SSIs come out as deep sternal wound infections and are often life-threatening involving frequently surgical redo (Filsoufi et al., 2009; Risnes et al., 2010). In current practice of medical microbiology, deep sternal wound infections is characterized by the isolation of one known opportunistic pathogen such as *Staphylococcus aureus*, Coagulase-negative Staphylococci (CoNS) and *Propionibacterium acnes* (Tammelin et al., 2002; Kühme et al., 2007; Risnes et al., 2010). These bacteria are generally considered to cause monomicrobial infections owing to their isolation in pure culture during the acute phase of cardiothoracic SSIs. However, the acute monomicrobial SSI might result in the disturbance of the operative site microbiota (Lu et al., 2013). Data about the dynamics of microbiota at the surgical site are currently scarce and worth to be described for a better understanding of SSIs pathophysiology. This study proposes the exploration of microbiota diversity at the sternal surgical site and its temporal variations during the surgical pathway of 25 patients undergoing coronary artery bypass graft (CABG).

## Materials and methods

### Patients, samples and ethic statements

Twenty-five patients who underwent CABG surgery at the service of Thoracic and Cardiovascular Surgery of the Montpellier University Hospital (France) in 2011 were included in a clinical study on SSI risk factors. For each patient, swabs were sampled before hospitalization, during intervention at surgical site superficially and deeply, and after intervention until the end of hospital stay (Supplementary Table 1). The first sample was taken on the skin of the sternum (C preop, for Cutaneous preoperative) before hospital admission to describe the patient normal microbiota. The second swab was sampled on the skin after antisepsis (C beginning) just before incision and the third one in sub-cutaneous tissue (SC beginning) once the incision is made. The fourth and fifth samples were done on sternum edges after sawing (SE beginning) and mediastinal tissue after positioning of sternal retractors (M beginning), respectively. At the end of operation, mediastinum (M end), sternum edges (SE end), sub-cutaneous tissue (SC end) and skin (C end) were sampled again according the step of surgical closure. At least, swabs were taken on surgical wound during dressing replacements, corresponding to skin and scar tissues sample (SST) (Supplementary Table 1).

Primary human materials used in this study were collected on sterile cotton swabs as performed for the routine clinical diagnostic process without change in the surgical procedures or the nursing cares. Each patient included gave an oral informed consent. The study proposal was approved by the ethical committee of our institution: Comité de Protection des Personnes Sud Méditerranée IV, N°ID—RCB: 2011-A00078-33 (Ref CPP: 11 02 02SC), and the collection of human samples was declared in the database “Conservation d'éléments du corps humain” at the French Ministry of Higher Education and Research (n° DC-2014-2169).

### 16S rRNA gene nested-PCR TTGE

Genomic DNA was directly extracted from cotton swabs by an enzymatic method (MasterPur Gram Positive DNA purification kit, EPICENTRE Biotechnologies®) as previously described (Romano-Bertrand et al., 2012). The 16S rRNA gene nested-PCR amplification was performed as previously described (Romano-Bertrand et al., 2012, 2014). Briefly, a fragment about 1465 bp of the 16S rRNA gene was amplified using the primers 27f and 1492r (Dekio et al., 2005). PCR products of this first amplification was used as template for the amplification of the 199-bp fragment (from position 338 to position 536, *Escherichia coli* numbering) overlapping the 16S rDNA V2-V3 variable region (Neefs et al., 1993; Sundquist et al., 2007), using the primers HDA1-GC (primer HDA1 with a fragment rich in GC—the “GC clamp”—added to the 59 extremity) and HDA2 (Ogier et al., 2002). The reaction mixture (50 μL) consisted of 200 nM of each primer (Sigma Genosys), 200 mM each dNTP (Fermentas), 2.5 U FastStart Taq DNA polymerase (Roche, France) in the appropriate reaction buffer, with 1.8 mM MgCl2. One microliter of DNA previously amplified was added to the reaction buffer and the thermal cycling was as follows: 95°C for 2 min; 35 cycles of 95°C for 1 min, 62°C for 30 s, 72°C for 1 min; and 72°C for 7 min. PCR products were checked by electrophoresis in a 1.5% agarose gel before TTGE migration.

TTGE migration was performed in the DCode universal Mutation Detection System (BioRad Laboratories). Gels were prepared with 8% (w/v) bisacrylamide (37.5:1), 7 M urea, 40 mL N,N,N9,N9-tetramethylethylenediamine, and 0.1% (w/v) ammonium persulfate, and were run in 1x Tris/Acetate/EDTA (TAE) buffer at pH 8.3. The electrophoresis conditions were a pre-migration for 15 min at 63°C and 20 V, followed by a migration at 46 V for 16 h with an initial temperature of 63°C and a final temperature of 70°C corresponding, to an increase of 0.4°C h^−1^ (Roudière et al., 2009).

Gels were stained for 15 min with 0.5 mg ethidium bromide mL^−1^ in 1x TAE buffer, washed for 45 min in 1xTAE buffer and photographed under UV transluminator.

### TTGE bands sequencing

For sequencing, each TTGE band was excised with disposable sterile scalpels to avoid contamination between bands, and washed 3 times in sterile distilled water. Gel slices were then incubated at 37°C overnight in 10 mM Tris buffer (pH 8.5) to allow DNA elution. Amplification of a single copy of the V2-V3 region of the 16S rRNA gene was performed using 1 μL of band eluate and the primers HDA1 without GC-clamp and HDA2. The PCR was carried out in 50 μL reaction mixture containing 200 nM of each primer, 200 nM each dNTP, 2.5 mM MgCl_2_ and 2.5 U Taq polymerase (Promega) in the appropriate buffer. PCR conditions were 94°C for 2 min; 35 cycles of 45 s at 95°C, 30 s at 62°C, 1 min at 72°C; and 10 min at 72°C. PCR products were checked by electrophoresis in a 1.5% agarose gel and sequenced on an ABI 3730xl sequencer (Cogenics, Beckman Coulter). Bands for which the sequencing process failed were analyzed by a new TTGE migration, as previously.

The sequences were checked for ambigous positions and analyzed by comparison with Genbank (http://www.ncbi.nlm.nih.gov/) and RDPII databases (http://rdp.cme.msu.edu/) using Basic Local Alignement Search Tool (BLAST) and Seqmatch programs, respectively. The RDPII database was particularly used for comparison to type strains of species. A sequence was affiliated to an Operational Taxonomic Unit (OTU) on the basis of a 99–100% of sequence identity (Drancourt et al., 2000; Jacquot et al., 2011). A sequence matching with the sequences of the type strain of only one validated species was affiliated to the corresponding taxonomic species. If the sequence matched at 99–100% identity with the sequences of several species of the same genus, the OTU was affiliated to the corresponding genus. If the best match was obtained with several genera in a familly or several famillies in an order, the OTU was affiliated to the corresponding familly and order, respectively and this, whatever the percent of identity.

### Analysis of TTGE fingerprints and diversity curves

An OTU was considered as present in a sample as soon as it was identified once. Diversity indexes (DI) were calculated by numbering the different OTUs detected in each sample, corresponding to the crude qualitative diversity. Rarefaction analysis was carried out using the online program *Analytic* available at http://strata.uga.edu/software/Software.html. The global diversity was classified in OTU groups of clinical or taxonomic relevance. The evolution of the different OTU groups according to the clinical course was represented by diversity curves. The importance of the qualitative and quantitative variations during the surgical pathway was assessed by the calculation of average rates and standard deviations for the different OTU groups during the hospitalization and graphically represented.

The methodology of samples analyzes is detailed in Supplementary Figure 1.

## Results

### Whole bacterial diversity at the surgical site

Two hundred ninety two cotton swabs were collected in 25 patients at 10 steps of their surgery and analyzed for bacterial diversity (Table 1). Three samples of the patient AH gave very faint signal in PCR and no visible pattern after TTGE. Direct sequencing succeeded for 86.4% of the sequenced bands. TTGE re-migration and re-sequencing increase the sequencing performance to 93.2%. A total of 1274 bands were affiliated to an OTU. The number of sequences for all samples per patient varied between 15 (for the patient EG) and 126 (for the patient AP), with an average of 51 sequences by patient. The overall number of sequences by type of sample was comprised from 98 for SC beginning to 219 for SST (Table 1). As awaited, we observed a decrease of sequence number between cutaneous sample before (C preop) and after asepsis (C beg). The decrease of sequences richness continued during the incision of sub-cutaneous tissus (SC beg) but re-increase in deeper samples. It was noteworthy that the sequences richness in the surgical wound was roughly similar with that of pre-operative cutaneous sample (Table 1).

**Table 1:** **Number of identified TTGE bands according to patient and intervention phase**.

**Patients**	**Preoperative (Preop)**	**Peroperative**								**Post-operative**	**All samples**
		**Beginning (beg)**				**End**					
	**C**	**C**	**SC**	**SE**	**M**	**M**	**SE**	**SC**	**C**	**SST**	
AB	7	3	2	2	4	9	5	5	2	5	44
AC	3	5	2	4	3	3	3	3	2	10	38
AD	3	1	2	3	5	7	4	7	5	7	44
AE	7	4	4	3	7	5	5	2	3	5	45
AF	2	2	13	6	4	9	7	7	5	5	60
AG	2	2	4	4	3	4	1	7	9	8	44
AH	6	5	1	2	1	0	0	2	2	9	28
AJ	2	5	6	4	5	5	5	4	3	13	52
AK	7	9	7	4	6	12	2	5	5	2	59
AL	10	16	3	5	5	6	4	4	3	12	68
AM	5	1	6	9	6	4	2	3	2	6	44
AP	6	MS	6	9	9	8	6	8	8	22	82
AQ	7	9	3	4	11	6	8	4	8	14	74
AR	7	5	3	6	12	5	1	9	3	MS	51
AS	8	4	4	4	6	3	6	4	5	11	55
AT	6	3	2	2	4	2	2	3	4	5	33
AW	9	4	3	4	5	6	2	4	2	3	42
AX	2	3	3	3	3	4	9	2	2	2	33
BB	5	6	2	2	8	6	1	3	7	7	47
BC	7	1	2	4	3	10	13	3	3	10	56
BD	8	10	4	7	4	10	16	7	13	47	126
CR	4	9	7	6	5	5	5	5	4	13	63
CV	6	4	2	4	6	6	5	3	6	3	45
DL	4	0	5	2	1	3	2	2	7	MS	26
EG	3	2	2	1	1	1	3	1	1	MS	15
All patients	136	113	98	104	127	139	117	107	114	219	1274

*C, cutaneous sample; SC, sub-cutaneous sample; SE, strenal edges sample; M, mediastinum sample; SST, skin and scar tissues sample; MS, missing sample*.

**Figure 1:**
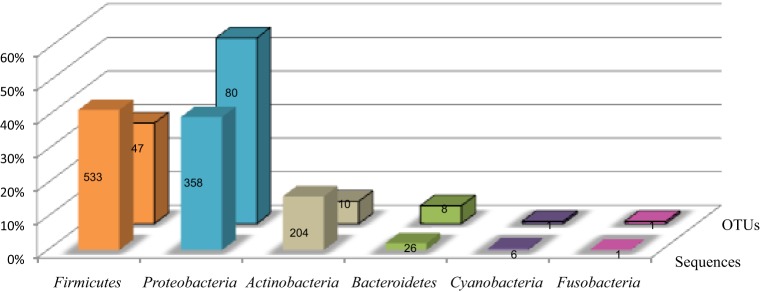
**Distribution of the TTGE bands sequences and OTUs by phylum**. The lane named “sequences” shows the percent of sequences affiliated to each phylum for a total of 1274 sequences. The lane named “OTUs” shows the percent of OTUs affiliated to each phylum for a total of 147 OTUs. The number of sequences and OTUs are given on each bar.

The sequences were distributed in 6 phyla as presented in Figure 1. *Firmicutes* and *Proteobacteria* were the major phyla with 41.7 and 39.7% of the sequences, respectively, beside *Actinobacteria* (16% of sequences), *Bacteroidetes* (2% of sequences), *Cyanobacteria* (0.5% of sequences) and *Fusobacteria* (0.1% of sequences) (Figure 1). The repartition of sequences according the type of cell wall structure was 57.7% for Gram-positive phyla and 42.3% for Gram-negative phyla.

**Figure 2:**
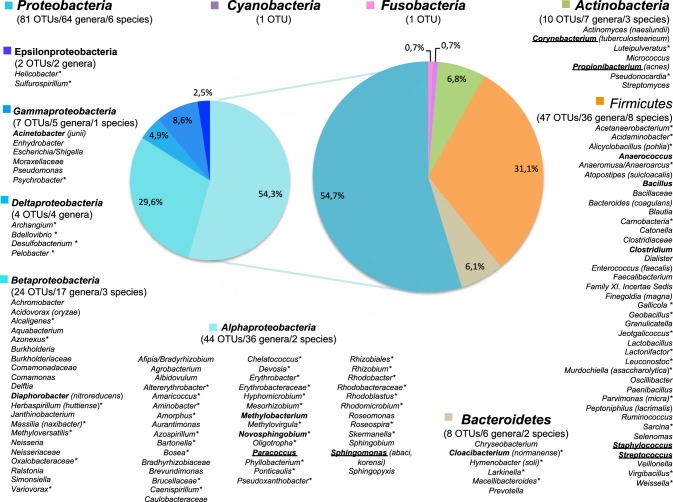
**The diversity of genus-level OTUs in surgical microbiota and their distribution by phylum**. The OTUs diversity is presented for all patient and samples. When available, the species affiliation is given in parenthesis. Genera in bold face were found in at least 30% of the patients and genera underlined were found in at least 50% of the patients. ^*^For OTUs never described in skin microbiota.

The 1274 TTGE bands were affiliated to 147 different OTUs. The OTU distribution by phylum for the whole patient's population is summarized in Figures 1, 2. The richness of the total bacterial communities was estimated by rarefaction analysis. The unsaturated shapes of the rarefaction curves indicated that bacterial richness was not yet completely sampled (Supplementary Figure 2). This confirmed the high bacterial diversity in the thoracic surgical site. More than half of OTUs belonged to *Proteobacteria* and one third to *Firmicutes*, the other phyla being minority. Among the *Proteobacteria*, *Alphaproteobacteria* was majority and represented about one third of the OTUs detected in this study (Figure 2). Fifty-seven OTUs (38.8%) corresponded to Gram-positive bacteria against 90 (61.2%) to Gram-negative bacteria. Therefore, in all patients and samples, bacteria belonging to Gram-positive phyla were more represented but less diverse than bacteria from Gram-negative phyla (Figures 1, 2).

Most OTUs (*n* = 112, 76.2%) were genus-level OTUs (Figure 2). High-quality sequences of the V3 region allowed accurate affiliation to the species level without ambiguities for 19 of the 147 OTUs (12.9%). The identified species are given in parenthesis in Figure 2. Conversely, some OTUs were affiliated only to a family (*n* = 13), an order (*n* = 1) or a phylum (*n* = 2).

The most prevalent genera in microbiota associated to thoracic surgery were *Staphylococcus* with 100% of the patients having at least one positive sample, *Propionibacterium* (88% of the patients), *Sphingomonas* (64% of the patients), and *Corynebacterium* (60% of the patients) (data not show). Nearly half of patients carried *Paracoccus* and *Streptococcus*. The carriages of *Anaerococcus* and *Acinetobacter* was detected in 40% of the patients and about one third of patient carried the genera *Bacillus*, *Clostridium*, *Methylobacterium*, *Novosphinogbium*, *Cloacibacterium* and *Diaphorobacter* (Figure 2). Finally, other OTUs were detected in less than a quarter of patients.

### Comparison of skin, wound and scar microbiota

**Figure 3:**
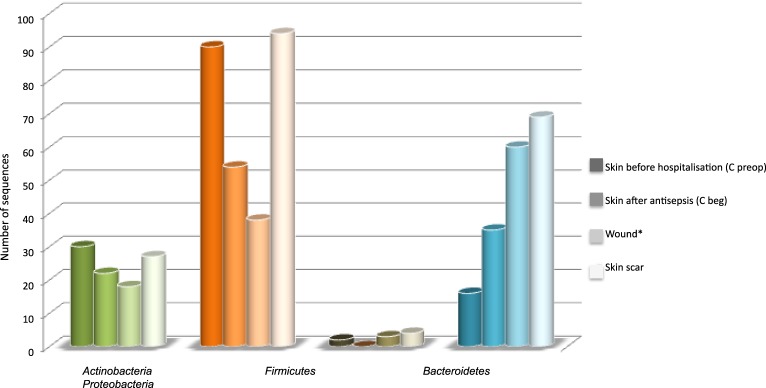
**Distribution of sequences according main phyla and types of sample**. ^*^The seven samples that corresponded to surgical wound were pooled and their diversity in sequence was expressed as the average of the seven samples in order to allow comparison with C preop, C beg and skin scar, which each corresponded to a single sample.

The distribution of 4 main phyla varied according the type of sample (Figure 3). Skin antisepsis provoked a marked decrease of *Firmicutes* in C beg samples and a lower decrease of *Actinobacteria* or *Bacteroidetes* compared to the skin before hospitalization (C preop). Considering that the effect of antisepsis on bacterial load is well known, this result confirmed that our approach was efficient to detected variations in skin microbiota. However, antisepsis led to an unexpected increase of *Proteobacteria* on the skin before surgical incision (Figure 3). Wound samples in Figure 3 corresponded to the pooling and averaging of the seven surgical steps for which the sub-cutaneous and deep tissus were open to outside. The trend of microbiota dynamics observed after antisepsis continued in the wound with a decrease of *Firmicutes* and *Actinobacteria* and an increase of *Proteobacteria* (Figure 3). In scar tissues, a trend to resilience regarding skin before hospitalization (C preop) was observed for *Firmicutes* and *Actinobacteria* but *Proteobacteria* remained markedly more prevalent than in C preop samples (Figure 3).

**Figure 4:**
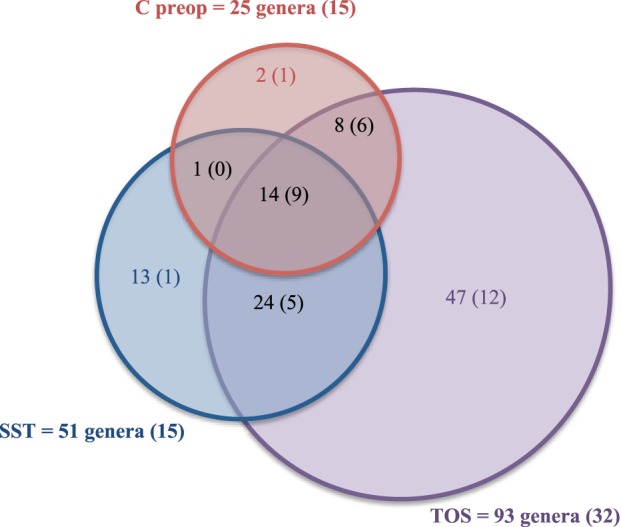
**Comparison of the diversity among preoperative skin, operative site and skin and scar tissues after surgery**. The numbers between parentheses correspond to the number of genera also identified in the human skin microbiota (Human Microbiome Project Consortium, 2012).

We therefore grouped C beg corresponding to skin samples after antisepsis but before incision with wound samples in order to represent the complete thoracic operative site (TOS) from pre-operative antisepsis to wound closure. The Figure 4 summarized the distribution of the 112 genera among the skin before hospitalization (C preop), the thoracic operative site (TOS) including skin after antisepsis and wound and the skin scar tissues (SST) microbiota. Comparison with the inventory of skin microbiota established in the HMP project (Human Microbiome Project Consortium, 2012) was also performed (Figure 4) (Supplementary Table 1). All the genera detected in skin samples before hospitalization (C preop) were also detected in TOS and SST except for *Veillonella* and *Pseudonocardia*, which were only found in the skin microbiota before hospitalization. Moreover, the 25 genera identified in C preop were also present in the HMP skin microbiota except *Pseudonocardia* (Supplementary Table 1). Both results suggested that the skin microbiota of the patient before surgical pathway presented no major particularities compared to the healthy human microbiota.

Fourteen genera were found together in C preop, TOS, and SST samples: Acidovorax, Acinetobacter, Anaerococcus, Bacillus, Clostridium, Corynebacterium, Diaphorobacter, Methylobacterium, Novosphingobium, Paracoccus, Propionibacterium, Sphingobium, Staphylococcus, and Streptococcus. Most of these core genera were found in at least one third of patients (12/14) as well as in the healthy skin microbiota defined in the HMP project (9/14). Among the 93 genera forming the TOS microbiota, only Propionibacterium, Staphylococcus and Sphingomonas were each shared by a majority of patients. These genera corresponded to the core^50^ TOS microbiota, i.e., the bacteria of the TOS microbiota shared by at least 50% of the patients at least in one sample. Beside these core OTUs, TOS microbiota displayed about 50% of specific OTUs (Supplementary Table 1). Thirty-four OTUs among them were undescribed in the HMP skin microbiota. These results suggested a high variability of the TOS microbiota among patients. The TOS panmicrobiota would probably increase with the number of patient and sample as suggested by the rarefaction curve obtained for 25 patients (Supplementary Figure 2).

It is important to note that 13 genera among the 51 detected in SST were specific to this site: *Archangium*, *Atopostipes*, *Caenispirillum*, *Carnobacterium*, *Gallicola*, *Luteipulveratus*, *Oligotropha*, *Rhodobacter*, *Sarcina*, *Virgibacillus*, and *Weissella* (Supplementary Table 1). They appeared very atypical compared to the known skin microbiota but all were patient-specific.

In conclusion, thoracic surgery and surgical wound healing led to the selection of atypical and specific taxa highly variable among patients beside core genera frequently found in skin human samples.

### Bacterial diversity and prevalence in patients during stages of surgery

**Figure 5:**
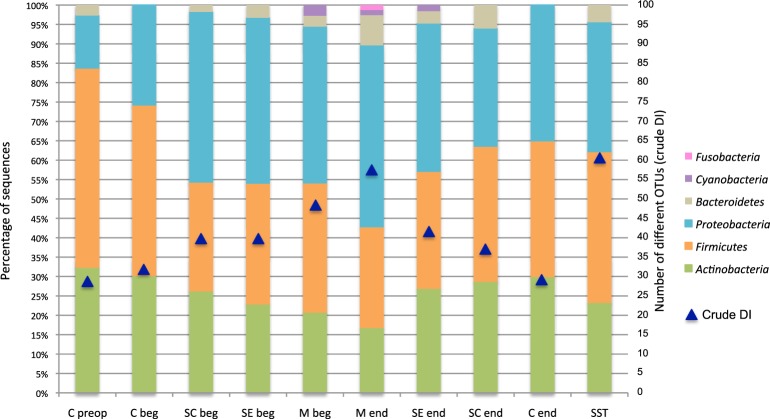
**OTUs diversity and percent of patients carrying each phylum according to the surgical stage**. Blue triangles display the evolution of the crude DI for all patients. Nomenclature of the stages is as described in Table 1: C for cutaneous, SC for sub-cutaneous, SE for sternal edges, M for mediastinum, beg and end for beginning and end of intervention respectively, preop for preoperative, SST for skin scar tissues.

The global diversity estimated by the crude diversity index (DI, corresponding to the number of different OTUs identified from all patients) at each stage of surgery is showed in the Figure 5. The DI was the lowest in skin before hospitalization (C preop), and the highest in skin scar (SST). It increased from 28 OTUs to 57 OTUs according to the surgical procedure and the depth of the sample between the C beg and M end (Figure 5). Then, it decreased in superficial samples at the end of the intervention during the wound closure procedure, from 57 OTUs in the M end to 29 OTUs on the skin at the end of the surgery (C end) (Figure 5). Finally, the crude DI increased again from 29 OTUs to 61 OTUs between the wound surgical closure and the scar tissues samples (Figure 5).

The microbiota dynamics to the phylum level according to the stage of surgery is summarized in the Figure 5, which indicated the percent of patients carrying each phylum. The phyla *Firmicutes* and *Proteobacteria* presented the most variable prevalence in patients according to operative stages. Globally, the carriage of Gram-positive bacteria (*Actinobacteria* and *Firmicutes*) was more frequent throughout the hospitalization pathway. However, it decreased in deep tissues (from 82% of patients in C preop to 43% in M end) (Figure 5). Conversely, the Gram-negative bacteria (*Proteobacteria*, *Bacteroidetes*, *Cyanobacteria*, and *Fusobacteria*) carriage increased with the depth of the sampling during the surgical intervention (Figure 5). The dynamics of bacterial diversity according to the surgical stage highlighted that the prevalence of *Firmicutes* and *Proteobacteria* evolved in a complementary manner.

**Figure 6:**
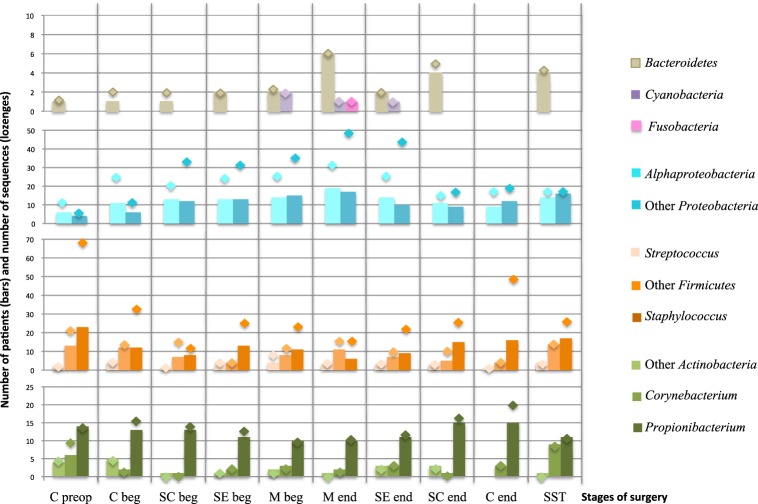
**Richness of OTUs groups and number of patients carrying each group according to the stage of surgery**. Bars chart corresponded to the number of patients carrying at least one representant of each OTU group. The lozenges represent the number of sequences obtained in each OTU group. Nomenclature of the stages is as described in Table 1: C for cutaneous, SC for sub-cutaneous, SE for sternal edges, M for mediastinum, beg and end for beginning and end of intervention respectively, preop for preoperative, SST for skin scar tissues. “Other *Proteobacteria*” for *Beta-, Gamma-, Delta*- and *Epsilon-Proteobacteria*; “Other *Actinobacteria*” for *Actinobacteria* but *Propionibacterium* and *Corynebacterium*; “Other *Firmicutes*” for *Firmicutes* but *Staphylococcus* and *Streptococcus*.

For further analysis, the 147 OTUs were classified in 11 groups. Because of their clinical interest in SSI, the genus *Staphylococcus*, *Propionibacterium*, *Streptococcus*, and *Corynebacterium* constituted four independent groups. The numbers of patients carrying each group are summarized in the Figure 6, which also give the richness in sequences retrieved for each OTU group (lozenges on the Figure 6). The importance of variations of the sequences richness and the taxonomic diversity during the whole surgical pathway is summarized in the Supplementary Figure 3. The number of patient with *Cyanobacteria* and *Fusobacteria* was low and did not vary significantly during the operation as confirmed by the stable number of sequence per OTUs (n≈1) (Supplementary Figure 3). *Bacteriodetes* varied with a punctual increase of diversity in the mediastinum at the end of the operation (M end).

The most variable OTU groups during the surgical course were: *Propionibacterium*, *Staphylococcus*, “Other *Firmicutes*” (excluding *Staphylococcus* and *Streptococcus*), *Alphaproteobacteria*, and “Other *Proteobacteria*” (excluding *Alphaproteobacteria*). The sequence richness curves for *Staphylococcus*, for *Alphaproteobacteria* and Other *Proteobacteria* largely exceeded the number of patient carrying these groups, i.e., each patient's positive sample contained more than one sequence of the group (Figure 6, Supplementary Figure 3). *Propionibacterium* varied with a slight decrease in frequency in deep samples and then a re-increase at the end of the intervention. Moreover, an increase of sequence richness in C end samples was observed. The prevalence of the *Proteobacteria* and *Staphylococcus* in patients during the operative pathway appeared complementary. *Proteobacteria* globally increased according to the surgery depth, with a peak in M end sample (49 and 31 TTGE bands corresponding to *Alphaproteobacteria* and Other *Proteobacteria* for 19 and 17 patients, respectively). In contrast with the proteobacterial groups, *Staphylococcus* was detected only for 6 patients corresponding to 17 TTGE bands at the same stage, instead of 23 patients and 70 sequences in the skin before intervention (Figure 6, Supplementary Figure 3). Therefore, the dynamics of *Staphylococcus* by itself explained most of the overall trend for Gram-positive bacteria observed in Figure 5.

**Figure 7:**
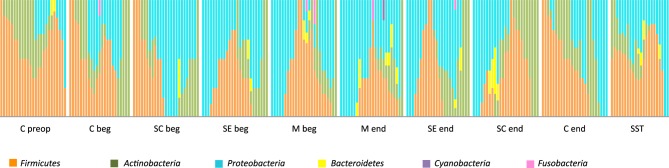
**Relative distribution of the 6 phyla in the 25 patients according to the stage of surgery**. Nomenclature of the stages is as described in Table 1: C for cutaneous, SC for sub-cutaneous, SE for sternal edges, M for mediastinum, beg and end for beginning and end of intervention respectively, preop for preoperative, SST for skin scar tissues.

When patients were individually considered as in Figure 7, we observed huge variations among patients for a single operative stage. However, the common traits of each stage remained visually obvious in Figure 7, particularly the shift toward Gram-negative during intervention and the only partial resilience of the original skin microbiota in healing tissues.

## Discussion

Many projects are devoted to human-associated microbiota in health and diseases. However, to our knowledge, none had already described the surgical site microbiota during operation, despite of the highly probable impact of the surgery on microbiota homeostasis and evidences for change in microbiota dynamics after barrier disruption (Zeeuwen et al., 2012). Microbiota equilibrium is challenged by numerous intrinsic factors (bacterial composition, host-cell interaction, host physiology, local immunity, among others) and environmental conditions. Any surgery provokes chemical aggressions by antisepsis, antimicrobial selective pressure by antibiotic prophylaxis as well as superficial or deep trauma with subsequent inflammation. CABG is a particularly invasive surgery because it needs full sternotomy, cardiopulmonary bypass, and cardioplegic arrest. The consequence is a local major disturbance associated to a systemic inflammation reaction that causes post-surgical morbidity including SSI (Filsoufi et al., 2001). Therefore, TOS microbiota during CABG appeared as a good model for a first description of the disturbance and dynamics of microbiota associated to surgery.

New Generation Sequencing (NGS) is becoming the state-of-the-art technology for the analysis of microbial communities from different ecosystems because an increase in sequence reads provides in-depth estimation of the biodiversity. Fingerprinting methods such as Denaturing Gradient Gel Electrophoresis (DGGE) or TTGE are classical methods consistent with NGS in respect to the detection of the predominant bacteria in human samples (Li et al., 2014; Nakatsu et al., 2014; Reen et al., 2014; Wacklin et al., 2014). Although fingerprinting revealed only OTUs with high abundance, this method is proven to be useful for comparison (Pires et al., 2012; Leite et al., 2014) and dynamics (Ding et al., 2012; Rungrassamee et al., 2013) of bacterial communities in different ecosystems. Moreover, community structure obtained from DGGE and TTGE data are shown to be in accordance with health status of the host (Santos and Bicalho, 2012; Li et al., 2014; Reen et al., 2014), physiology (Wacklin et al., 2014), diet (Nakatsu et al., 2014), life-style (Bangsgaard et al., 2012), biogeography (Morrow et al., 2012), and niche (Pires et al., 2012; Leite et al., 2014). In some studies based on both fingerprinting and NGS, DGGE or TTGE seemed more clearly related to pathological, physiological or ecological conditions (Morrow et al., 2012; Santos and Bicalho, 2012). One hypothesis is that the detection of the huge diversity of minority taxa by NGS might blur correlation observed with majority taxa. Recent studies that used both approaches recognized that they give correlated and complementary information: DGGE or TTGE are good methods for the survey of communities and their variations according time or conditions, and NGS allows in-depth description of the communities with the exhaustive identification of their minor bacterial components.

We choose 16S rRNA PCR-TTGE for the follow-up and the comparison of TOS microbiota of 25 patients across 10 steps of their surgical pathway. Due to the low load of bacteria in surgical wound, amplification was performed by nested-PCR as previously described for community with low richness before either fingerprinting (Ding et al., 2012; Pires et al., 2012; Santos and Bicalho, 2012; Li et al., 2014) or pyrosequencing (Pires et al., 2012; Reen et al., 2014). Nested-PCR before denaturing fingerprint has been described as a robust, reliable and sensitive tool for the analysis of skin bacteria community structure (Li et al., 2014) and allowed a preliminary detection of surgical wound bacteria (Romano-Bertrand et al., 2014).

During operation, the surgical site is considered as quasi-sterile although the microbiota present in deep tissues were rarely studied. In routine, sampling for microbiological analysis of the surgical site is performed only when the surgeon detected infection symptoms. Besides, many procedure are performed with the aim to limit bacterial contamination in the surgical site: antisepsis and antibiotic prophylaxis for the patient, wound edge protection, surgery team attire, operating room air treatment and surfaces cleaning, improved practices in anaesthesiology and surgery, etc (Beckmann et al., 2011). Published research studies on the surgical site contamination are based on culture and they remain scarce mainly because no clear correlation was found with the occurrence of SSI (Bouza et al., 2006).

One major result of this study is that the surgical site presents a huge bacterial diversity from the incision step to the closure, even in deep mediastinal samples. As for all DNA-based approaches of the biodiversity, the detection of DNA is not synonymous of presence living organisms. However, the particular dynamics of surgical microbiota with Gram-negative shift in the intervention followed by the partial resilience of the preoperative microbiota during healing suggested the description of a living system differing to DNA contamination. We detected a total of 147 OTUs in all samples and patients, most being identified to the genus-level. As no study of the operative microbiota is available, we compare our results with previous findings on skin microbiota. The high species richness in TOS microbiota is unexpected. For comparison, the HMP identified 87 OTUs on 4 different skin areas from 24 adults, (Human Microbiome Project Consortium, 2012).

TOS OTUs belongs to the 6 phyla *Proteobacteria*, *Firmicutes*, *Actinobacteria*, *Bacteroidetes*, *Fusobacteria* and *Cyanobacteria*, which was globally in accordance with the phylum diversity described in skin microbiota studies (Dekio et al., 2005; Grice et al., 2008, 2009; Human Microbiome Project Consortium, 2012; Zeeuwen et al., 2012; Li et al., 2014). However, the repartition of the different phyla in the surgical microbiota differed comparing to the microbiome composition of different skin sites (Grice and Segre, 2011). It is noteworthy that the repartition of skin microbiota is highly site-dependant with high influence of the microenvironment of the site (local humidity, presence of sebaceous glands…) (Grice et al., 2009; Grice and Segre, 2011; Zeeuwen et al., 2012) and the sternum skin area has not been studied for healthy microbiota so far. We found a majority of OTUs that belong to *Proteobacteria* followed by *Firmicutes*, *Bacteroidetes* and *Actinobacteria*. The *Proteobacteria* phylum was the most diverse with 80 OTUs including 42 OTUs never yet described in skin microbiota (Dekio et al., 2005; Gao et al., 2007; Grice et al., 2009; Human Microbiome Project Consortium, 2012; Zeeuwen et al., 2012). When we consider the sequence abundance, *Firmicutes* and *Proteobacteria* are equally represented followed by *Actinobacteria* and *Bacteroidetes*. This *Proteobacteria*-dominant type of microbiota does not correspond to any other described skin microbiota but *Proteobacteria*-rich sites was found mainly along the arm from the axillary vault to the interdigital space but also in the buttock (Grice and Segre, 2011). The previously studied skin zone mostly related to sternum is manubrium, which is quasi exclusively colonized by *Corynebacterium*, *Propionibacterium* and *Micrococcus* (Grice and Segre, 2011). In sternal swabs (C preop) sampled before the patient hospitalization without previous skin antisepsis, we detected *Corynebacterium*, *Propionibacterium* and *Micrococcus* as described for manubrium site in healthy skin but also a great diversity of *Firmicutes* and *Proteobacteria* with a quantitative dominance of *Staphylococcus*.

As described for many human-associated microbiota and especially for the skin microbiota, the inter-individual variation of the surgical microbiota was very high, as only 8 OTUs were shared by almost 40% of the patients (Human Microbiome Project Consortium, 2012). And the TOS microbiota was characterized by its own diversity compared to the preoperative skin and skin and scar tissues, with 50% of TOS-specific genera.

*Staphylococcus* population decreases during the operation, which is probably related to the antimicrobial effect of antiseptics and antibiotics given as SSI prophylaxis (Mangram et al., 1999). Antibiotic prophylaxis is focused on the main species involved in cardiothoracic SSIs, i.e., *Staphylococcus aureus*, coagulase negative staphylococci and *Propionibacterium acnes*. The decrease of staphylococcal sequences during the intervention and their increase at the end suggested that antibiotics are efficient on *Staphylococcus* populations. The same effect is observed for *P. acnes* but to a lesser extent, partially because its persistence on the skin despite the preoperative skin preparation (Lee et al., 2014). The *P. acnes* species is sub-divided in diverse phylotypes, the phylotype IB increase during cardiothoracic operation in spite of antibiotic prophylaxis (Romano-Bertrand et al., 2014). This particular behavior of *P. acnes* phylotype IB can explain the only slight decrease observed in this study for the genus *Propionibacterium* during the operative steps covered by antibiotic prophylaxis. *Staphylococcus* and *Propionibacterium* are less represented in scar skin than in preoperative healthy skin, suggesting that the resilience of the skin microbiota is not complete or takes more time than explored herein. Indeed, studies demonstrated the long-term microbiota disturbance after antibiotic treatment (Dethlefsen et al., 2007; Jernberg et al., 2007). A decrease between one-fourth to one-third of the microbial diversity in the digestive tract (Dethlefsen et al., 2007; Jernberg et al., 2007) persisted for 2-years post-treatment (Jernberg et al., 2007). Short-term effect 1 week after treatment is also observed with paradoxical maintenance or increase of bacterial load and the selection of Gram-negative over Gram-positive bacteria (Panda et al., 2014). We observed a similar change of Gram-negative/Gram-positive ratio between preoperative samples and perioperative or post-operative samples. Beside antimicrobial agents, skin and tissue disruption is a major stress in surgery. Experimental barrier disruption demonstrated that the healing neo-microbiome was more similar to that of the deeper stratum corneum layers than to the initial surface microbiome (Zeeuwen et al., 2012). Herein, the healing tissues are colonized by a neo-microbiota that differed clearly from the preoperative skin microbiota but more related to surgical site microbiota. One can hypothesizes that bacteria selected in deep skin layers and tissues during surgery recolonize the healing surface in place to previous resident bacteria.

During the operation, the increase in diversity and richness of Gram-negative bacteria (*Proteobacteria* and *Bacteroidetes*) according the depth of sampling site deserves to be discussed. It is complementary to the *Staphylococcus* and *Propionibacterium* decreases. Most Gram-negative genera corresponded mainly to bacteria known to be environmental. The *Alphaproteobacteria* diversity is particularly important in TOS microbiota and illustrated the relationships between taxonomic diversity in TOS and in diverse environment. Numerous genera of TOS *Alphaproteobacteria* are classically found in marine water, wastewater, and polluted soil, or in symbiosis with plants. Surprisingly, several genera are phototrophic: *Rhodobacter*, *Rhodoblastus*, *Rhodomicrobium*, *Roseospira*, and *Skermanella* in *Rhodobacterales*. Some of environment-associated genera are more and more described in opportunistic human infections. For instance, it is the case for some species and genotypes in the genera *Aurantimonas* (Luong et al., 2008), *Agrobacterium* (Aujoulat et al., 2011), *Methylobacterium* (Kovaleva et al., 2014), *Paracoccus* (Schweiger et al., 2011), *Roseomonas* (Bard et al., 2010), and *Sphingomonas* (Cheong et al., 2008). The sequence affiliation in *Betaproteobacteria* and *Deltaproteobacteria* also displays environment-associated genera, only some of them being involved in human infections: *Achromobacter*, *Burkholderia*, and *Neisseria*. *Gammaproteobacteria* in TOS are less diverse and corresponded mostly to genera involved in human infections. Indeed, beside *Bacteroides* and *Prevotella* in *Bacteroidetes*, *Acinetobacter*, *Pseudomonas*, and *Escherichia* are the sole well-known Gram-negative opportunistic pathogens involved in HAI and other infections detected herein. These genera were also involved in cardiac SSI (Gårdlund et al., 2002; Bouza et al., 2006; George et al., 2006; Chaudhuri et al., 2012). Finally, 55% of the bacterial genera detected in TOS (including 70% for *Alphaproteobacteria*) were never detected in skin microbiota.

Conversely, a majortity (74%) of Gram-positive bacteria detected herein were also found in the previously described skin microbiota (Dekio et al., 2005; Gao et al., 2007; Grice et al., 2009; Human Microbiome Project Consortium, 2012) and other human microbiota (Human Microbiome Project Consortium, 2012). The Gram-positive microbiota is dominated by anaerobes that belong to human oral and gut microbiota (Human Microbiome Project Consortium, 2012). These bacteria have been previously described in human infection and sometimes in endocarditis and other cardiovascular infections. Finally, major pathogens involved in cardiac SSIs were also detected: *Staphylococcus*, *Propionibacterium*, *Streptococcus* and *Enterococcus* (Filsoufi et al., 2009).

In a genus, pathogenicity and life-style are often species-dependent but metagenomic community inventories are generally limited to a genus affiliation. We affiliated some high-quality sequences of *Firmicutes* and *Actinobacteria* to a recognized pathogenic species involved in cardiac SSIs: *Propionibacterium acnes* (Romano-Bertrand et al., 2014), *Corynebacterium tuberculostearicum* (Hinic et al., 2012), *Finegoldia magna* (Fournier et al., 2008), and *Enterococcus faecalis* (Bouza et al., 2006). Other species are known to provoke other types of human infection: *Actinomyces naeslundii*, *Parvimonas micra*, *Murdochiella asaccharolytica*, *Peptoniphilus lacrimalis*, and *“Bacteroides” coagulans*. Other species-affiliated sequences corresponded to bacteria never yet described in human-associated samples. Except *Alicyclobacillus pohliae* and *Atopostipes suicloacalis* from *Firmicutes*, they belong to Gram-negative phyla. *Hymenobacter soli* and *Cloacibacterium normanense* correspond to the *Bacteroidetes* phylum. *Shingomonas abaci*, *Sphingomonas koreensis*, *Acidovorax oryzae*, *Diaphorobacter nitroreducens*, and *Herbaspirillum huttiense* belong to the *Proteobacteria*. The sole proteobacterial species known as opportunistic pathogen is *Acinetobacter junii*.

Finally, we describe in TOS a complex bacterial community that gathers pathobionts and bacteria deemed to be environmental, opportunistic pathogens and non-pathogenic bacteria. These data stress to consider TOS microbiota as a “pathobiome” (Vayssier-Taussat et al., 2014) rather than a reservoir of individual potential pathogens. The new paradigm of pathobiome may be applied to most opportunistic infections and mainly to HAI but it needs to change conventional practices in medical microbiology based mainly on the isolation of microorganisms in pure culture as well as on the consideration of only well-known pathogens as the cause of infection. Caracterization of whole microbial community is currently incompatible with the urgency of SSI diagnosis but samples could be retrospectively studied to the community level in order to describe bacteria associated to the main pathogen that succeeded in SSI. This step of clinical microbiology on acute SSI samples appears necessary to link TOS microbiota and SSI. In our prospective study, this link could not be done. Indeed, due to the low incidence of SSI (3% in our hospital) the number of patients necessary to be included would be high and incompatible with in the depth microbiota description.

The pathobiome-based concept of infection collides with methodological barriers and for instance, in this study, the description of TOS microbiota raises more questions than provides answers about SSI mechanisms. As for most metagenomic studies, the species richness and variability among subjects impaires the data interpretation and the most significant microbiological variations are seen for high taxonomic groups such as phyla. Interpretation concerning skin microbiota in health and disease used a “cutaneotype” based on the global Gram-positive/Gram-negative ratio (Alekseyenko et al., 2013). Herein, we observe that this ratio varies also along the surgical pathway. We also simplify the huge diversity by the description of a core TOS microbiota. Pending appropriate models and methods that take into account the complete diversity, phyla ratio and core genera should be already confronted to further clinical studies in order to link these potential markers to the clinical status of the patient and its evolution. Prospective studies on large cohorts are requiered to determine if these markers are predictive of SSIs. Moreover, the patient's risk factors of SSI in cardiac surgery are well known: age, sex, active smoking, diabetes mellitus, obesity, ASA score… In further studies, the TOS dynamics will be compared to patient's risk factors in order to determine if the link between clinical risk factor and SSI occurence involved TOS dynamics.

### Conflict of interest statement

The authors declare that the research was conducted in the absence of any commercial or financial relationships that could be construed as a potential conflict of interest.
