# Supplementary material for: Dynamics of the surgical microbiota along the cardiothoracic surgery pathway
Source: Front Microbiol. 2015 Jan 13;5:787. doi: 10.3389/fmicb.2014.00787 (PMC4292786; doi:10.3389/fmicb.2014.00787)
Supplement: Supplementary file 1 [file SupplementaryMaterials.DOCX]

***Supplementary material***

**Dynamics of the surgical microbiota along the cardiothoracic surgery pathway**

Romano-Bertrand Sara^1,2^, Jean-Marc Frapier^3^, Brigitte Calvet^4^, Philippe Colson^3^, Bernard Albat^3^, Parer Sylvie^1,2^ & Jumas-Bilak Estelle^1,2^

^1^ Equipe Pathogènes et Environnements, UMR 5119 ECOSYM, Université Montpellier 1, Montpellier, France

^2^ Département d’Hygiène Hospitalière, Centre Hospitalier Régional Universitaire de Montpellier, Montpellier, France

^3^ Service de Chirurgie Thoracique et Cardiovasculaire, Centre Hospitalier Régional Universitaire de Montpellier, Montpellier, France

^4^ Service de Réanimation de Chirurgie Cardiothoracique, Centre Hospitalier Régional Universitaire de Montpellier, Montpellier, France

*** Correspondence: Dr. Sara Romano-Bertrand, Université Montpellier 1, UFR Pharmacie, UMR 5119 ECOSYM, Equipe Pathogènes et Environnements, 15 avenue Charles Flahault BP14491, 34095 Montpellier Cedex 5, France sara.romano-bertrand@univ-montp1.fr**

**Supplementary Table 1.** Comparison of genera present on the skin before hospitalization (C preop), in the thoracic operative site (TOS) including skin after antisepsy and wound, and in the skin scar tissues (SST). Skin microbiota described in the Human Metagenome Project (skin HMP) was also compared. The common genera between the 4 microbiota are in bold type. ^§^Genera identified only in the preoperative skin microbiota (C preop). *Genera identified only in the TOS microbiota. ^#^Genera identified only in the skin and scar tissues microbiota (SST). °Genera identified only in the skin microbiota by the Human Microbiome Preoject (HMP).

| **All identified genera** | **C preop** | **TOS** | **SST** | **Skin HMP** |
| --- | --- | --- | --- | --- |
| *Abiotrophia°* |  |  |  | X |
| *Acetanaerobacterium** |  | X |  |  |
| *Achromobacter** |  | X |  |  |
| *Acidaminobacter** |  | X |  |  |
| *Acidovorax* | X | X | X |  |
| ***Acinetobacter*** | **X** | **X** | **X** | **X** |
| *Actinobacillus°* |  |  |  | X |
| *Actinomyces* |  | X |  | X |
| *Afipia* |  | X |  | X |
| *Agrobacterium** |  | X |  |  |
| *Albidovulum** |  | X |  |  |
| *Alcaligenes** |  | X |  |  |
| *Alicyclobacillus** |  | X |  |  |
| *Alistipes°* |  |  |  | X |
| *Altererythrobacter** |  | X |  |  |
| *Amaricoccus* |  | X | X |  |
| *Aminobacter* |  | X | X |  |
| *Amorphus* |  | X | X |  |
| ***Anaerococcus*** | **X** | **X** | **X** | **X** |
| *Anaeromusa/Anaeroarcus** |  | X |  |  |
| *Anaerostipes°* |  |  |  | X |
| *Anhycrobacter°* |  |  |  | X |
| *Aquabacterium** |  | X |  |  |
| *Archangium^#^* |  |  | X |  |
| *Atopobium°* |  |  |  | X |
| *Atopostipes ^#^* |  |  | X |  |
| *Aurantimonas** |  | X |  |  |
| *Azonexus** |  | X |  |  |
| *Azospirillum** |  | X |  |  |
| ***Bacillus*** | **X** | **X** | **X** | **X** |
| *Bacteroides* |  | X | X | X |
| *Barnesiella°* |  |  |  | X |
| *Bartonella** |  | X |  |  |
| *Bdellovibrio** |  | X |  |  |
| *Bifidobacterium°* |  |  |  | X |
| *Blautia* |  | X |  | X |
| *Bosea* |  | X | X |  |
| *Brachybacterium°* |  |  |  | X |
| *Bradyrhizobium* |  | X |  | X |
| *Brevundimonas* |  | X |  | X |
| *Burkholderia* |  | X | X | X |
| *Butyricicoccus°* |  |  |  | X |
| *Butyricimonas°* |  |  |  | X |
| *Caenispirillum^#^* |  |  | X |  |
| *Campylobacter°* |  |  |  | X |
| *Capnocytophaga°* |  |  |  | X |
| *Carnobacteria^#^* |  |  | X |  |
| *Catenibacterium°* |  |  |  | X |
| *Catonella* |  | X |  | X |
| *Chelatococcus** |  | X |  |  |
| *Chryseobacterium* |  | X | X |  |
| *Cloacibacterium* |  | X | X |  |
| ***Clostridium*** | **X** | **X** | **X** | **X** |
| *Collinsella°* |  |  |  | X |
| *Comamonas* |  | X | X |  |
| *Coprobacillus°* |  |  |  | X |
| *Coprococcus°* |  |  |  | X |
| ***Corynebacterium*** | **X** | **X** | **X** | **X** |
| *Cronobacter°* |  |  |  | X |
| *Delftia* |  | X |  | X |
| *Desulfobacterium** |  | X |  |  |
| *Devosia** |  | X |  |  |
| *Dialister* | X | X |  | X |
| *Diaphorobacter* | X | X | X |  |
| *Dolosigranulum°* |  |  |  | X |
| *Dorea°* |  |  |  | X |
| *Enhydrobacter** |  | X |  |  |
| *Enterococcus* |  | X | X |  |
| *Erythrobacter* |  | X | X |  |
| *Escherichia* |  | X |  | X |
| *Eubacterium°* |  |  |  | X |
| *Faecalibacterium* |  | X |  | X |
| *Finegoldia* | X | X |  | X |
| *Fusobacterium°* |  |  |  | X |
| *Gallicola^#^* |  |  | X |  |
| *Gardnerella°* |  |  |  | X |
| *Gemella°* |  |  |  | X |
| *Geobacillus** |  | X |  |  |
| *Granulicatella* |  | X |  | X |
| *Helicobacter* | X | X |  |  |
| *Herbaspirillum** |  | X |  |  |
| *Holdemania°* |  |  |  | X |
| *Hymenobacter* |  | X | X |  |
| *Hyphomicrobium** |  | X |  |  |
| *Janthinobacterium* |  | X | X |  |
| *Jeotgalicoccus* |  | X | X |  |
| *Kingella°* |  |  |  | X |
| *Lactobacillus* | X | X |  | X |
| *Lactococcus°* |  |  |  | X |
| *Lactonifactor** |  | X |  |  |
| *Larkinella** |  | X |  |  |
| *Leptotrichia°* |  |  |  | X |
| *Leuconostoc** |  | X |  |  |
| *Luteipulveratus^#^* |  |  | X |  |
| *Macellibacteroides* | X | X |  |  |
| *Massilia (Naxibacter)* |  | X | X |  |
| *Megasphaera°* |  |  |  | X |
| *Mesorhizobium** |  | X |  |  |
| *Methylobacterium* | X | X | X |  |
| *Methyloversatilis** |  | X |  |  |
| *Methylovirgula** |  | X |  |  |
| *Micrococcus* | X | X |  | X |
| *Mitsuokella°* |  |  |  | X |
| *Mogibacterium°* |  |  |  | X |
| *Murdochiella* |  | X | X |  |
| *Neisseria* |  | X |  | X |
| *Novosphigobium* | X | X | X |  |
| *Odoribacter°* |  |  |  | X |
| *Oligotropha^#^* |  |  | X |  |
| *Oscillibacter* |  | X |  | X |
| *Paenibacillus* | X |  | X |  |
| *Parabacteroides°* |  |  |  | X |
| *Paracoccus* | X | X | X |  |
| *Paraprevotella°* |  |  |  | X |
| *Parasutterella°* |  |  |  | X |
| *Parvimonas** |  | X |  |  |
| *Pasteurella°* |  |  |  | X |
| *Pelobacter** |  | X |  |  |
| *Pelomonas°* |  |  |  | X |
| *Peptinophilus* |  | X | X | X |
| *Phascolarctobacterium°* |  |  |  | X |
| *Phenylobacterium°* |  |  |  | X |
| *Phyllobacterium** |  | X |  |  |
| *Ponticaulis** |  | X |  |  |
| *Porphyromonas°* |  |  |  | X |
| *Prevotella* | X | X |  | X |
| ***Propionibacterium*** | **X** | **X** | **X** | **X** |
| *Providencia* |  | X |  | X |
| *Pseudomonas* |  | X |  | X |
| *Pseudonocardia^§^* | X |  |  |  |
| *Pseudoxanthobacter** |  | X |  |  |
| *Psychrobacter* |  | X | X |  |
| *Ralstonia* |  | X | X | X |
| *Rhizobium** |  | X |  |  |
| *Rhodobacter^#^* |  |  | X |  |
| *Rhodoblastus** |  | X |  |  |
| *Rhodomicrobium** |  | X |  |  |
| *Roseburia°* |  |  |  | X |
| *Roseomonas* |  | X | X |  |
| *Roseospira* |  | X | X |  |
| *Rothia°* |  |  |  | X |
| *Ruminococcus* | X | X |  | X |
| *Sarcina^#^* |  |  | X |  |
| *Selenomonas* |  |  | X | X |
| *Simonsiella* |  | X | X |  |
| *Skermanella** |  | X |  |  |
| *Sneathia°* |  |  |  | X |
| ***Sphingobium*** | **X** | **X** | **X** | **X** |
| *Sphingomonas* |  | X | X | X |
| *Sphingopyxis** |  | X |  |  |
| *Sporacetigenium°* |  |  |  | X |
| ***Staphylococcus*** | **X** | **X** | **X** | **X** |
| *Stenotrophomonas°* |  |  |  | X |
| ***Streptococcus*** | **X** | **X** | **X** | **X** |
| *Streptomyces* | X | X |  |  |
| *Streptophyta°* |  |  |  | X |
| *Subdoligranulum°* |  |  |  | X |
| *Sulfurospirillum** |  | X |  |  |
| *Suttleworthia°* |  |  |  | X |
| *Tannerella°* |  |  |  | X |
| *Treponema°* |  |  |  | X |
| *Variovorax* |  | X | X |  |
| *Veillonella* | X |  |  | X |
| *Virgibacillus^#^* |  |  | X |  |
| *Weissella^#^* |  |  | X |  |
| *Yersinia°* |  |  |  | X |
| Total | 26 | 94 | 51 | 87 |
| Number of orphans | 2 | 35 | 12 | 51 |

**Supplementary Figure 1. Schematic representation of community fingerprint by TTGE.** A and B represented two different samples. Step 1: The total bacterial DNA was directly extracted from the cotton swabs by an enzymatic method. Step 2: The 16S-rRNA genes of the bacterial community were amplified by nested-PCR. Step 3: The 16S-rDNA amplicons were separated by TTGE according to their nucleotidic sequences. Step 4: The isolated TTGE bands were affiliated to an Operational Taxonomic Unit (OTU) by sequencing and comparison with Genbank (http://www.ncbi.nlm.nih.gov/) and RDPII databases (http://rdp.cme.msu.edu/) using Basic Local Alignement Search Tool (BLAST) and Seqmatch programs, respectively.

Supplementary Figure 2. Rarefaction curve for all samples from 25 patients.

**Supplementary Figure 3: Variations of the taxonomic diversity (OTUs) and the sequences richness for the 11 OTUs groups for all patients and samples.** The figure is based on the average rates and standard deviations of the DI (i.e. number of OTU) and the richness in sequences corresponding to the different OTU groups. The top lines represent the variation of the richness in sequences corresponding to each group during the hospitalization. The bottom lines represent the variation of the number of OTU corresponding to each group during the hospitalization. The boxplot represents the shared area of variation between the number of OTU and the richness in sequences: a black boxplot means overlapping variations of the numbers of OTU and sequences richness, whereas white boxplot represents non- overlapping variations.
